# *Wolbachia*-Mitochondrial DNA Associations in Transitional Populations of *Rhagoletis cerasi*

**DOI:** 10.3390/insects11100675

**Published:** 2020-10-05

**Authors:** Vid Bakovic, Martin Schebeck, Christian Stauffer, Hannes Schuler

**Affiliations:** 1 Department of Forest and Soil Sciences, University of Natural Resources and Life Sciences Vienna, BOKU, Peter-Jordan-Strasse 82/I, A-1190 Vienna, Austria; martin.schebeck@boku.ac.at (M.S.); christian.stauffer@boku.ac.at (C.S.); 2 Faculty of Science and Technology, Free University of Bozen-Bolzano, Universitätsplatz 5, I-39100 Bozen-Bolzano, Italy; hannes.schuler@unibz.it

**Keywords:** *Wolbachia*, cytoplasmic incompatibility, bacterial spread, imperfect maternal transmission, field populations, transition zones

## Abstract

**Simple Summary:**

*Wolbachia* is an endosymbiotic bacterium that infects numerous insects and crustaceans. Its ability to alter the reproduction of hosts results in incompatibilities of differentially infected individuals. Therefore, *Wolbachia* has been applied to suppress agricultural and medical insect pests. The European cherry fruit fly, *Rhagoletis cerasi*, is mainly distributed throughout Europe and Western Asia, and is infected with at least five different *Wolbachia* strains. The strain *w*Cer2 causes incompatibilities between infected males and uninfected females, making it a potential candidate to control *R. cerasi*. Thus, the prediction of its spread is of practical importance. Like mitochondria, *Wolbachia* is inherited from mother to offspring, causing associations between mitochondrial DNA and endosymbiont infection. Misassociations, however, can be the result of imperfect maternal transmission, the loss of *Wolbachia*, or its horizontal transmission from infected to uninfected individuals. These are important parameters influencing the spread of infection. Here, we studied *Wolbachia*-mitochondrial haplotype associations in *R. cerasi* in two transition zones in the Czech Republic and Hungary, where *w*Cer2 is currently spreading. Our results suggest imperfect maternal transmission only in the early phases of *w*Cer2 invasion and no evidence of horizontal transmission of *w*Cer2 in *R. cerasi*.

**Abstract:**

The endosymbiont *Wolbachia* can manipulate arthropod host reproduction by inducing cytoplasmic incompatibility (CI), which results in embryonic mortality when infected males mate with uninfected females. A CI-driven invasion of *Wolbachia* can result in a selective sweep of associated mitochondrial haplotype. The co-inheritance of *Wolbachia* and host mitochondrial DNA can therefore provide significant information on the dynamics of an ongoing *Wolbachia* invasion. Therefore, transition zones (i.e., regions where a *Wolbachia* strain is currently spreading from infected to uninfected populations) represent an ideal area to investigate the relationship between *Wolbachia* and host mitochondrial haplotype. Here, we studied *Wolbachia*-mitochondrial haplotype associations in the European cherry fruit fly, *Rhagoletis cerasi*, in two transition zones in the Czech Republic and Hungary, where the CI-inducing strain *w*Cer2 is currently spreading. The *w*Cer2-infection status of 881 individuals was compared with the two known *R. cerasi* mitochondrial haplotypes, HT1 and HT2. In accordance with previous studies, *w*Cer2-uninfected individuals were associated with HT1, and *w*Cer2-infected individuals were mainly associated with HT2. We found misassociations only within the transition zones, where HT2 flies were *w*Cer2-uninfected, suggesting the occurrence of imperfect maternal transmission. We did not find any HT1 flies that were *w*Cer2-infected, suggesting that *Wolbachia* was not acquired horizontally. Our study provides new insights into the dynamics of the early phase of a *Wolbachia* invasion.

## 1. Introduction

*Wolbachia* are a group of maternally inherited Alphaproteobacteria that infect a wide range of nematode and arthropod species [1,2]. These endosymbionts influence the reproduction of their hosts and enhance their own spread. The most common phenotype in insects is cytoplasmic incompatibility (CI), which results in embryonic death when the sperm of an infected male fertilizes the egg of an uninfected female (unidirectional CI) or a female infected with another incompatible strain (bidirectional CI) [3]. *Wolbachia* is currently being used as a novel tool to control insect pest species by repressing populations due to CI and by reducing the ability to replicate pathogens in insect vector species [4,5,6,7,8,9]. Analogous to the sterile insect technique (SIT), where laboratory-reared males are sterilized prior to field releases [4], the incompatible insect technique (IIT) is based on the release of *Wolbachia*-infected incompatible males, which will lead to the suppression of natural pest populations. Nevertheless, models based on attributes of the strain and its host are necessary to predict the spread of the endosymbiont in field populations (e.g., [10,11]).

*Wolbachia*’s ability to induce unidirectional CI renders infected female hosts a reproductive advantage compared to uninfected females because infected females produce viable offspring by mating with both infected and uninfected males, whereas uninfected females only produce viable offspring by mating with uninfected males [3]. Thus, the frequency-dependent reproductive advantage of infected females results in the spread of the CI-inducing *Wolbachia* strain. Furthermore, the maternal transmission of the endosymbiont causes selective sweeps of associated host mitochondrial haplotypes [12,13]. Associations between *Wolbachia* strain and mitochondrial haplotype of its host have been studied in numerous insect species (e.g., [14,15,16,17]), including several drosophilid and tephritid fruit fly systems [12,18,19,20,21]. From these studies, almost perfect associations have been shown to result from high levels of CI-induction and nearly complete maternal transmission of *Wolbachia*, even when small amounts of paternal and/or horizontal transmission are present [12]. Misassociations between *Wolbachia* strain and host mitochondrial haplotype, however, have been found and can result from factors such as imperfect maternal transmission, loss of *Wolbachia* infection, and intra- and interspecific horizontal transmission [18,22]. The relationship between *Wolbachia* and host mitochondrial haplotype in field populations can therefore provide insights into the dynamics of an ongoing *Wolbachia* invasion [23].

The dynamics of *Wolbachia* and host mitochondrial haplotype are most pronounced and best studied in ongoing selective sweeps of *Wolbachia* strains through natural populations (e.g., [24,25]). Diffusion equations have been used to depict *Wolbachia* transition zones as traveling waves, characterized by transects consisting of high to low infection frequencies [10,24,25,26,27].

The European cherry fruit fly, *Rhagoletis cerasi* (Linnaeus, 1758), is particularly well suited for the study of *Wolbachia* spread. This tephritid fly infests cherries (*Prunus* spp.) and honeysuckle (*Lonicera* spp.) and is mainly distributed throughout Europe and western Asia [28,29]. *Rhagoletis cerasi* is infected with at least five different *Wolbachia* strains, *w*Cer1-5, whose spread is traceable on small spatial scales due to the fly’s univoltine life cycle and poor dispersal [30,31,32,33]. The CI-induction of *w*Cer2 best explains previously reported incompatibilities among European populations of *R. cerasi* [34] and was further shown to induce CI when transferred into *Drosophila simulans* [35] and *Ceratitis capitata* [36]. Although *w*Cer4 also showed evidence of CI-induction when transferred into *C. capitata* [36], it is less clear as to what phenotypes it, and other *w*Cer strains, cause in *R. cerasi*.

European-wide surveys of *w*Cer2 and *R. cerasi* mitochondrial haplotypes revealed the presence of two mitochondrial haplotypes, HT1 and HT2, across various European populations [18,36]. In *w*Cer2-fixed regions, all flies are HT2, while in uninfected regions, all flies are HT1 [18]. Misassociations between *w*Cer2 and HT2 were only found in the vicinity of transition zones (i.e., the forefronts of *w*Cer2 spread where both haplotypes and infection types can be found) [18,36]. These consist of both *w*Cer2-infected flies associated with HT1 and *w*Cer2-uninfected flies associated with HT2. The former is best explained by intraspecific horizontal transmission from *w*Cer2-infected to uninfected *R. cerasi* individuals, while the latter can arise from imperfect maternal transmission of *w*Cer2, wherein an infected female transmits to offspring its mitochondrial haplotype but not its infection type [18,23].

In Germany, *w*Cer2 infection frequencies follow a clear pattern, spreading from the north and south into the central region, which is *w*Cer2-uninfected. Further collections of *R. cerasi* over smaller spatial scales and over multiple years showed that in transition zones *w*Cer2 does not follow a typical gradient from high to low infection frequencies. Instead, a scattered *w*Cer2-infection pattern was found [18,36]. In contrast to these findings, *w*Cer2 transition zones found in the Czech Republic and Hungary show wave-like smooth gradients from high to low infection frequencies [33]. In the Czech Republic, the transition from *w*Cer2-fixed to *w*Cer2-uninfected populations was described within a 46-km transect from south to north. The *w*Cer2 transition zone in Hungary showed a gradual decrease from *w*Cer2-fixed to low infection frequencies within a 72-km west to east transect [33].

In this study, we determined the mitochondrial haplotypes of *R. cerasi* field populations used in [33] and characterized the *w*Cer2-mitochondrial haplotype associations in these two transition zones. As *w*Cer2 is perfectly associated with HT2 in populations completely invaded by *w*Cer2, misassociations are only found in transitional populations and can be considered transient [18], representing a window in time to study the dynamics of *w*Cer2 and its associated mitochondrial haplotype in a natural setting. The *w*Cer2 transition zones in the Czech Republic and Hungary are unique in that they represent clear invasion fronts with smooth infection gradients [33]. This allows the study of *w*Cer2 spread dynamics and its associated haplotypes in natural field populations.

## 2. Materials and Methods

**Figure 1:**
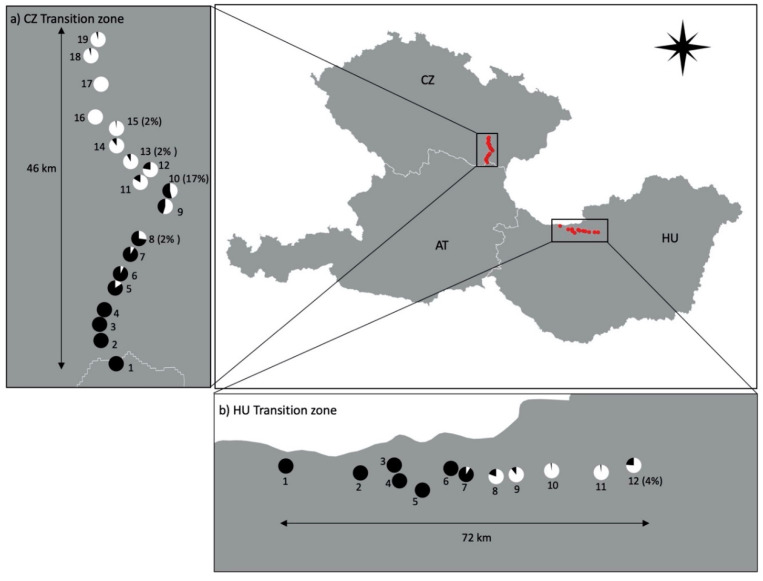
The distribution of *Rhagoletis cerasi* mitochondrial haplotypes, HT1 and HT2, in two *w*Cer2 transition zones within the Czech Republic (CZ), Hungary (HU) and Austria (AT). White and black pie-charts denote HT1 and HT2 frequencies, respectively. Percentages in parentheses indicate frequencies of misassociated HT1 flies infected with *w*Cer2.

In total, 881 *R. cerasi* samples were collected in the Czech Republic (CZ) and Hungary (HU), whose *w*Cer2 infection frequencies have been previously published [33]. Here, we determined the mitochondrial haplotypes of these *R. cerasi* samples and their associations with *w*Cer2. Each population was sampled from a single cherry tree. In transition zone CZ, 545 individuals were collected from 19 populations (CZ-1 to CZ-19). In transition zone HU, 336 individuals were collected from 12 populations (HU-1 to HU-12) (Figure 1).

The mitochondrial haplotype of *R. cerasi* individuals was determined using PCR-RFLP (Restriction Fragment Length Polymorphism), as described by [17]. Briefly, a 546-bp fragment of the mitochondrial COI gene was amplified using the primers Pat and Dick [37]. The two mitochondrial haplotypes of *R. cerasi* are distinguished by a single nuclear polymorphism (SNP). RFLP-haplotype determination was performed by incubating 0.5 µL of the PCR product with 0.5 U of *HaeIII* (Thermo Fisher Scientific, Waltham, MA, USA) under 37 °C for 4 h and loaded on a 2% agarose gel. After incubation with *HaeIII*, haplotype HT2 is cut into a 342- and 204-bp fragment, while haplotype HT1 remains undigested (Supplementary Figure S1) [17].

The mitochondrial PCR products from a portion of samples from CZ populations, including individuals that showed *Wolbachia*-haplotype misassociations, were confirmed by Sanger sequencing. Single strand sequencing of the COI fragment from samples showing the *Wolbachia*-haplotype misassociations was performed by Eurofins MWG Operon (Ebersberg, Germany). Sequence chromatograms were first inspected in ChromasLite version 2.1. (Technelysium, Brisbane, Australia), and subsequently edited in GeneRunner version 5.0. (www.generunner.net). Finally, the sequences were determined using the BLAST algorithm [38]. Information on individual haplotypes, the method used to determine the haplotypes, and Sanger sequences are found in Supplementary Table S1.

## 3. Results

In the majority of cases, *w*Cer2-infected *R. cerasi* flies were associated with mitochondrial haplotype HT2, while *w*Cer2-uninfected flies were associated with mitochondrial haplotype HT1. Out of the 881 flies analyzed, we found seven HT2 flies that were *w*Cer2-uninfected and no HT1 flies that were *w*Cer2-infected. Six of these misassociations were found within transitional populations, having both infected and uninfected individuals, whereas one individual of HT2 was found within a *w*Cer2-uninfected population (CZ-15). In the Czech transition zone, around 1.1% of the individuals within transitional populations were *w*Cer2-uninfected but linked with HT2. Similarly, around 0.8% of the individuals in Hungarian transitional populations were HT2 whilst being *w*Cer2-uninfected (Figure 1).

In the Czech Republic, all flies from *w*Cer2-fixed populations (i.e., CZ-1 to CZ-4) were assigned the mitochondrial haplotype HT2. The association of HT2 with *w*Cer2 was perfect in populations with high *w*Cer2 infection frequencies. To the contrary, misassociations were found in populations with low *w*Cer2 infection frequencies. In total, five *Wolbachia*-haplotype misassociations were found: one in CZ-8, two in CZ-10, one in CZ-13, and one in CZ-15 (Figure 1).

In Hungary, all flies from *w*Cer2-fixed populations (HU-1 to HU-6) were assigned the mitochondrial haplotype HT2. Two *w*Cer2-uninfected flies were linked to HT2 and were found in HU-12 (Figure 1).

## 4. Discussion

The smooth gradient from high to low *w*Cer2 infection frequencies within the Czech and Hungarian *Wolbachia* transition zones represent a hotspot to study the dynamics of an ongoing *Wolbachia* spread. Here, we focused on the association between *w*Cer2 infection and the *R. cerasi* mitochondrial haplotype to understand the dynamics of an ongoing selective sweep of *Wolbachia*. In contrast to the scattered *w*Cer2 infection pattern previously studied in German transition zones [18,36], we can now identify in which part of the wave *Wolbachia*-mitochondrial haplotype misassociations are more likely to occur [33]. In accordance with the pattern observed in German *w*Cer2 transition zones [18,36], we found *w*Cer2-uninfected individuals associated with HT2 within populations with low *w*Cer2 infection rates. The occurrence of *w*Cer2-uninfected individuals associated with HT2 in *w*Cer2-fixed populations was not found. Surprisingly, we detected HT2 even in one population that was not infected by *w*Cer2 (CZ-15). This suggests that imperfect *Wolbachia* transmission or occasional losses of *Wolbachia* occur in the early phase of invasion in *R. cerasi*. Moreover, the absence of *w*Cer2-infected HT1 flies suggests no evidence for intraspecific horizontal transmission, which contrasts previous findings in Germany [18,36].

The maternal transmission rate varies among host organisms and *Wolbachia* strains. Examples of high *Wolbachia* transmission rates include *w*Ri of *D. simulans*, where transmission rates reach 95.2% [39], and in *w*Au of *D. simulans* the rate is estimated at 97.7% [25]. Similarly, we found that populations of *R. cerasi* show a high rate of maternal transmission through high *Wolbachia*-mitochondrial haplotype association rates, further supporting the presence of high CI induction. A perfect association between *w*Cer2 and HT2 was found within populations outside the transition zone, highlighting the *Wolbachia*-driven selective sweep of HT2. However, within transitional populations with a low *w*Cer2 infection frequency, we found *w*Cer2-uninfected individuals associated with HT2. This indicates that imperfect maternal transmission and/or occasional loss of *Wolbachia* is occurring in the early phase of *Wolbachia* invasion. In transitional and *w*Cer2-uninfected populations, females might be able to pass the misassociation to their offspring by mating with uninfected males. The likelihood of mating with uninfected males will decrease in populations with high *w*Cer2 infection rates. Mating of *w*Cer2 uninfected females with *w*Cer2-infected males will result in embryonic mortality due to CI and will therefore be a dead end for individuals uninfected with *w*Cer2 but associated with HT2 as *w*Cer2 approaches fixation.

*Wolbachia* can be horizontally transferred from infected to uninfected individuals both within species [18] and between species [22,40]. Findings from German field populations of *R. cerasi* showed a substantial amount of *w*Cer2-infected HT1 flies, an indication of intraspecific horizontal transmission [18,36]. In contrast, no *w*Cer2-infected HT1 flies were found in this study. Currently, we can only speculate about the discrepancy of intraspecific horizontal transmission rates between *w*Cer2 transition zones found in Germany and those found in the Czech Republic and Hungary. Schuler et al. [18] reported a higher incidence of *w*Cer2-infected HT1 flies collected on honeysuckle as compared to flies collected from cherry. Since all flies analyzed in this study were collected from cherries, the host might play a crucial role. Different fruit sizes (honeysuckle being significantly smaller than cherries) might increase the likelihood of cannibalism by co-occurring larvae infesting the same fruit. Moreover, parasitoids may transfer *w*Cer2 from infected to uninfected flies [41,42]. Since parasitation rates may vary between host forms with differentially sized host fruit [43,44,45], a lower parasitation rate in cherry-infesting flies might reflect the low number of intraspecific horizontal *Wolbachia* transfer in cherry flies.

Our results add on to a list of studies that highlight the problem of relying on only mitochondrial barcoding for demographic and population history inference in arthropod systems [13], as *Wolbachia*-driven selective sweeps of mitochondrial haplotypes will produce misleading results. Recent advances in genome sequencing [46] provide a great opportunity to reveal novel insights into the dynamics of this early-phase *Wolbachia* invasion on a genomic level. Based on the genome sequence data from [47], the genomic architecture of the CI trait in *w*Cer2 [48] will help to reveal the genetic basis of CI in this naturally multiple-infected host and the interaction between different *Wolbachia* strains [49]. Finally, genome sequencing of the fruit fly within transitional populations will help to reveal if the invading *Wolbachia* strain leaves (transitional) footprints on the fly genome.

## 5. Conclusions

The *w*Cer2 transition zones in the Czech Republic and Hungary present an excellent region to study the association of a spreading *Wolbachia* strain and its co-inherited mitochondrial haplotype. Here, we describe an almost strict association of *w*Cer2 with mitochondrial haplotype HT2, with a few cases where HT2 flies were *w*Cer2-uninfected. These misassociations indicate that imperfect maternal transmission occurs in early phases of *Wolbachia* invasion where they can further spread, but these misassociations are transient and will be eliminated as *w*Cer2 approaches fixation. Future time-series studies on the co-inheritance of *w*Cer2 and HT2 in these transition zones will provide a source of empirical data to test models describing the dynamics of *Wolbachia*-haplotype associations within an invasion front in natural populations.

## Supplementary Materials

The following are available online at https://www.mdpi.com/2075-4450/11/10/675/s1: Table S1: Method used for mitochondrial haplotype determination for each *Rhagoletis cerasi* individual accompanied by the Sanger sequence where applicable; Figure S1: Electrophoresis gel showing an example of HT1 and HT2 digestion with restriction enzyme *HaeIII*.
