# Supplementary material for: Wolbachia-Mitochondrial DNA Associations in Transitional Populations of Rhagoletis cerasi
Source: Insects. 2020 Oct 5;11(10):675. doi: 10.3390/insects11100675 (PMC7650823; doi:10.3390/insects11100675)
Supplement: Supplementary file 1 [file insects-11-00675-s001.zip › insects-931589-supplementary materials/Figure S1.pdf]

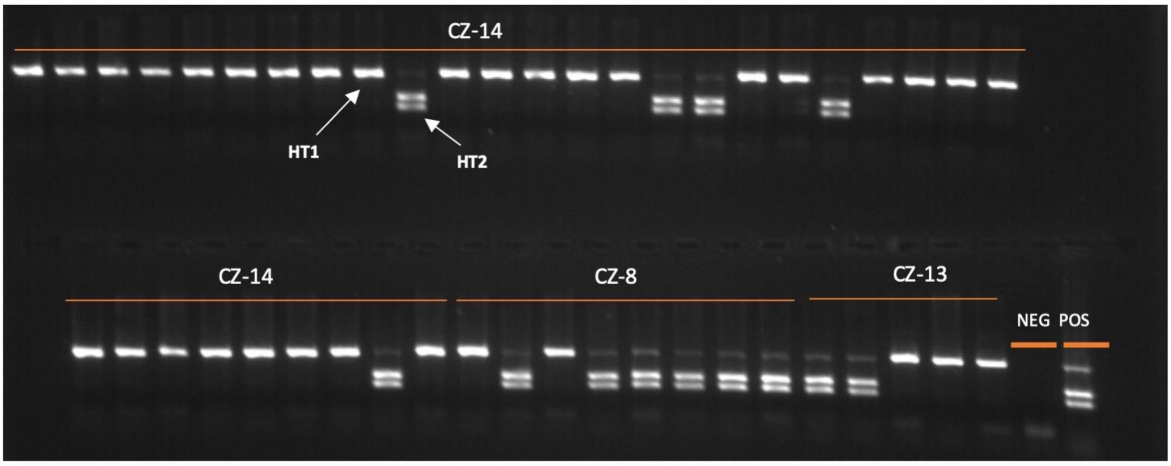

Figure S1. Electrophoresis gel showing an example of HT1 and HT2 digestion with restriction enzyme *HaeIII*.
